# Unraveling Non‐Uniform Strain‐Induced Crystallization Near a Crack Tip in Natural Rubber

**DOI:** 10.1002/advs.202307741

**Published:** 2024-01-16

**Authors:** Thanh‐Tam Mai, Tomohiro Yasui, Ruito Tanaka, Hiroyasu Masunaga, Taizo Kabe, Katsuhiko Tsunoda, Shinichi Sakurai, Kenji Urayama

**Affiliations:** ^1^ Department of Material Chemistry Graduate School of Engineering Kyoto University Nishikyo‐ku Kyoto 615‐8510 Japan; ^2^ Department of Biobased Materials Science Kyoto Institute of Technology Kyoto 606‐8585 Japan; ^3^ Japan Synchrotron Radiation Research Institute Sayo‐gun Hyogo 679‐5198 Japan; ^4^ Sustainable and Advanced Materials Division Bridgestone Corporation Tokyo 187‐8531 Japan

**Keywords:** crystallization, elastomer, natural rubber, rubber elasticity, synchrotron radiation

## Abstract

Strain‐induced crystallization (SIC) in natural rubber (NR) near crack tips significantly enhances crack growth resistance, but understanding the interplay between local strain field and crystallization remains challenging due to confined and heterogeneous characteristics. Using micro‐scale digital image correlation (DIC) and scanning wide‐angle X‐ray diffraction (WAXD, with a narrow 10 µm square beam), this study maps local strain tensor properties and SIC in the vicinity of the crack tip and its peripheral zone (≈3 mm × 1 mm area). The analysis reveals a significant correlation between these properties. In the peripheral zone, there is a noticeable deviation of both the principal strain axis and the crystal orientation from the crack opening direction. These deviations are linearly correlated, which indicates that shear strain plays a significant role in determining the crystal orientation. Crucially, the maximum tensile component in the tensor of local principal strains predominantly dictates local crystallinity. This simplicity is attributed to the limited variation in types of deformation within the SIC region, with corresponding to deformations falling between planar and uniaxial stretching. These findings pave the way for predicting crystallinity distribution using solely strain field data, offering valuable insights into the role of SIC in enhancing the crack growth resistance of NR.

## Graphical abstract

This study explores the interplay between non‐uniform strain and crystallization near crack‐tips in natural rubber, employing micro‐scale DIC and micro‐beam scanning X‐ray scattering. Local maximum tensile strain predominantly dictates local crystallinity, while shear strain governs crystal orientation. These findings shed light on how crystallization contributes to the crack growth resistance of natural rubber.

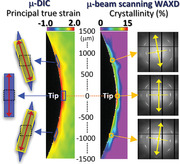

## 1 Introduction

Rubber, an essential cornerstone of modern life, has applications spanning diverse products, from tires to gloves and sports equipment.^[^ ^1^, ^2^, ^3^ ^]^ Natural rubber (NR), derived from latex of the Para rubber tree, stands in contrast to synthetic rubber that has its roots in petroleum sources.^[^ ^4^, ^5^, ^6^ ^]^ Despite being integral to industries for over a century, the significance of NR is accentuated in today's era, promoting recycling and sustainability.^[^ ^7^, ^8^ ^]^ Beyond its bio‐based origin, a distinctive feature of NR is its propensity for strain‐induced crystallization (SIC) – a unique phenomenon where pronounced strain leads to partial crystallization of NR molecules.^[^ ^9^, ^10^, ^11^, ^12^, ^13^, ^14^, ^15^, ^16^, ^17^ ^]^ This crystallization significantly stiffens the rubber, introducing a marked increase in tensile stress at high elongation. As a result, NR exhibits heightened tensile strength and fracture toughness, functioning as its own reinforcement mechanism.^[^ ^18^, ^19^ ^]^ The latent heat release during SIC of NR has potential for applications like soft, biodegradable solid‐state elastocaloric cooling devices.^[^ ^20^ ^]^ These distinctive features place NR a cut above other rubbers devoid of the SIC capability. Furthermore, it has been observed that gels with high solvent concentrations also exhibit SIC, markedly enhancing their mechanical toughness.^[^ ^21^, ^22^ ^]^ SIC is a crucial influencer in augmenting the toughness of polymer‐based soft materials, spanning both rubbers and gels.

While numerous studies have examined SIC in NR, focusing on its interplay with variables like strain and temperature, most have employed unnotched tensile specimens subjected to uniform uniaxial stretching.^[^ ^9^, ^10^, ^11^, ^12^, ^23^, ^24^, ^25^, ^26^ ^]^ SIC commences rapidly, initiating within 100 milliseconds following the application of strain.^[^ ^27^, ^28^, ^29^ ^]^ Crystallization then proceeds incrementally even as stress diminishes, given a maintained constant strain.^[^ ^30^, ^31^, ^32^ ^]^ These results suggest that strain is a more pivotal factor in the crystallization process than stress. The reinforcing capability of SIC also substantially contributes to enhancing the tear strength and suppressing the catastrophic crack growth.^[^ ^33^, ^34^, ^35^, ^36^, ^37^, ^38^, ^39^, ^40^, ^41^, ^42^ ^]^ In general, the strain near a crack tip in rubber is markedly non‐uniform, while it steeply increases with approaching the crack tip.^[^ ^43^, ^44^, ^45^, ^46^, ^47^, ^48^, ^49^ ^]^ In proximity to the crack tip of NR, the localized strain intensifies sufficiently to trigger SIC.^[^ ^12^, ^37^, ^50^, ^51^, ^52^, ^53^ ^]^ The resistance to crack growth empowers materials to endure significant loads, even in the presence of an initial crack, thus boosting their durability. However, characterizing SIC around a crack front is challenging owing to its localized and non‐uniform nature. Prior work, employing wide‐angle X‐ray diffraction (WAXD) with a narrow incident beam, confirmed the occurrence of SIC near crack tips in pre‐notched NR samples under both static^[^ ^52^, ^53^ ^]^ and dynamic^[^ ^37^, ^50^, ^51^ ^]^ loads. These studies showed that the SIC region expands several hundred micrometers from the crack tip in the neat and carbon‐black filled NR specimens.^[^ ^37^, ^50^, ^51^, ^52^, ^53^ ^]^ They reported that a decrease in crosslink density expanded the SIC zone, while a rise in temperature or an increasing thermal aging time led to a reduction in crystallinity around a crack tip. Some researchers^[^ ^12^, ^37^, ^50^ ^]^ utilized digital image correlation (DIC)^[^ ^54^ ^]^ to characterize the non‐uniform strain field surrounding the crack tip, debating the relationship with the spatial distributions of SIC properties. Yet, a holistic understanding of these correlations remains elusive due to the inherent confined and non‐uniform characteristics of the SIC around a crack tip.

Our study embarks on this exploration, leveraging a potent combination of micro‐beam WAXD and micro‐scale DIC techniques —both having superior spatial resolution than in previous studies. Utilizing a 2D WAXD scan with a 10 µm square beam at 40 µm intervals over a wide area of ≈3 mm × 1 mm, we can discern SIC occurrences and quantify the crystallinity distribution exceedingly close to the stationary crack contour in NR specimens. Complementarily, the micro‐DIC method offers high‐resolution mapping of local strain tensor via optical microscopy observation of fine speckle patterns on the specimen surface. The speckles typically measure 20 µm in size, and the image process captures these patterns at a high resolution, with each pixel on the camera sensor corresponding to ≈4 µm on the specimen. This combined analysis provides a robust foundation for elucidating the interplay between the non‐uniform SIC and strain fields in stationary cracks. We delve into various SIC properties such as the spatial distributions of crystallinity and crystal orientation near a crack tip and relate them to properties of the local strain tensor. Importantly, our approach unravels a governing quantity in the local principal strain tensor that influences local SIC in both the vicinity of the crack tip and its peripheral zone. This result enriches our understanding of the SIC behavior near a crack tip in strain crystallizing rubber. It further hints at the potential to infer the spatial distribution of crystallinity at any degree of crack tip opening, using solely the associated strain field data, without the need for the data of the spatial distribution of crystal diffraction.

## 2 Results and Discussion

**Figure 1:**
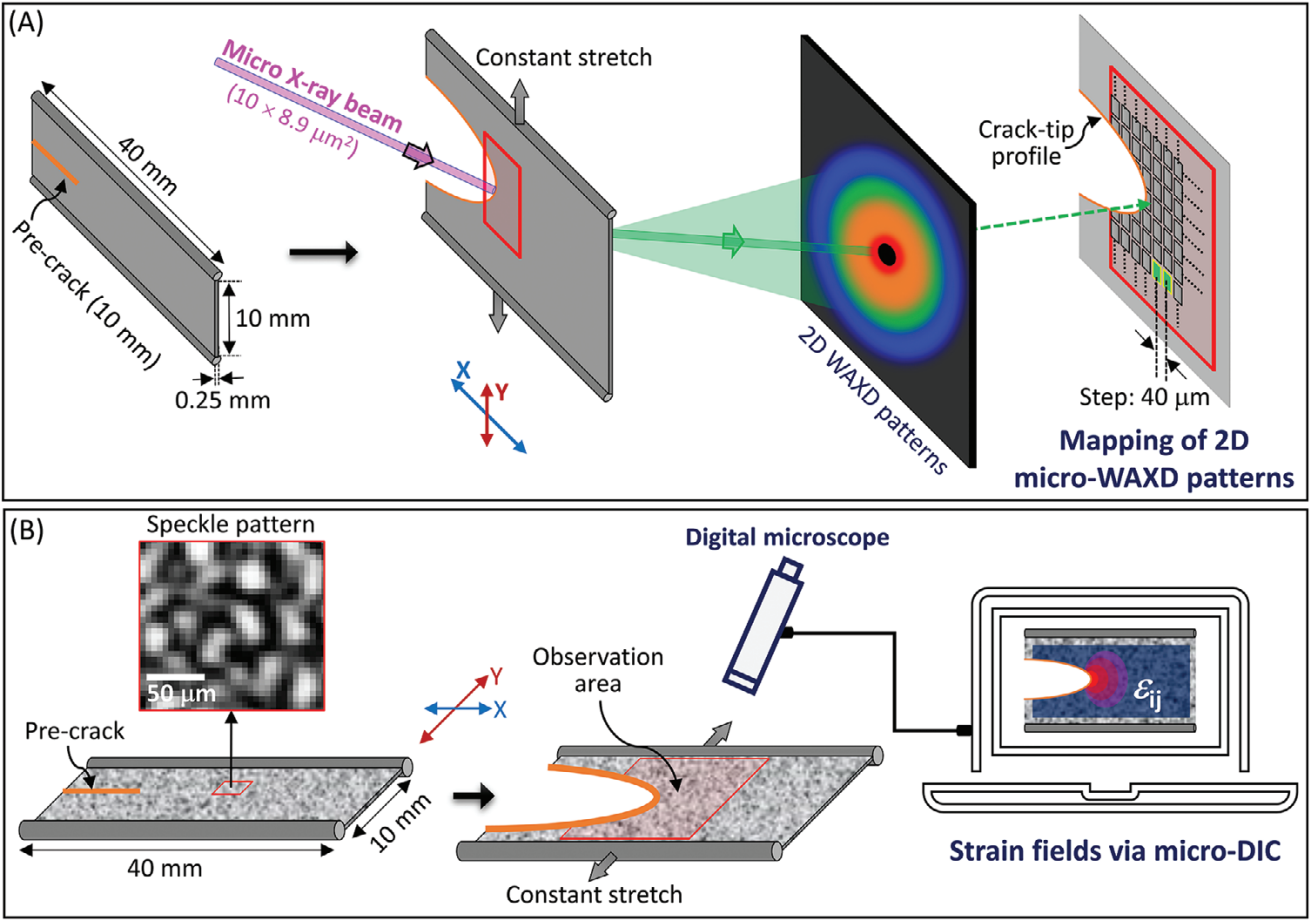
Complementary analysis using 2D scanning micro‐beam WAXD and micro‐DIC measurements for SIC near a crack tip in a NR. A) Schematic of the real‐time scanning WAXD using a narrow 10 µm square beam with a 0.118 nm wavelength. The scan grid around the crack tip consists of 21 points on the X‐axis and 75 points on the Y‐axis, spaced 40 µm apart. B) Schematic of the micro‐DIC (digital image correlation) setup used for 2D local strain tensor measurement. Micro‐speckle images, with a pixel size of ≈4 µm, were captured by using a digital microscope. Both micro‐beam WAXD and micro‐DIC techniques were applied to the exact same specimen, enabling precise data correlation. Measurements were conducted after the equilibration under a constant tensile true strain of 0.62 (or nominal strain of 0.85). Prior to the measurements, the specimen underwent several preliminary loading‐unloading cycles to exclude the strain‐hysteresis effect on stress, which is detailed in Experimental Section.

SIC and strain field in the vicinity of a crack tip and its peripheral zone of NR have been elucidated through micro‐beam WAXD (**Figure** **1**A) and micro‐DIC (Figure 1B) methodologies, respectively. Each method is detailed in Materials and Methods Section. The measurements were carried out on a stationary crack in a wide NR sheet specimen (40 mm × 10 mm × 0.25 mm) featuring a 10 mm single‐edge notch along the long (X‐) axis and subjected to a macroscopic true tensile strain (*ε*_Y,macro_) of 0.62 (or nominal strain of 0.85) along the short (Y‐) axis. The applied tensile strain, while being below the critical strain required for crack growth initiation, is sufficient to promote SIC around the crack tip. The present issue can be treated as a 2D problem due to the sufficiently thin film geometry. To precisely correlate the results of the micro‐beam WAXD and micro‐DIC measurements, we utilized the exact same specimen and an identical custom‐made tensile instrument in both sets of tests. Prior to the measurements, the specimen was subjected to preliminary loading‐unloading cycles which was designed to remove strain‐hysteresis effect on stress, which was detailed in Experimental Section.

**Figure 2:**
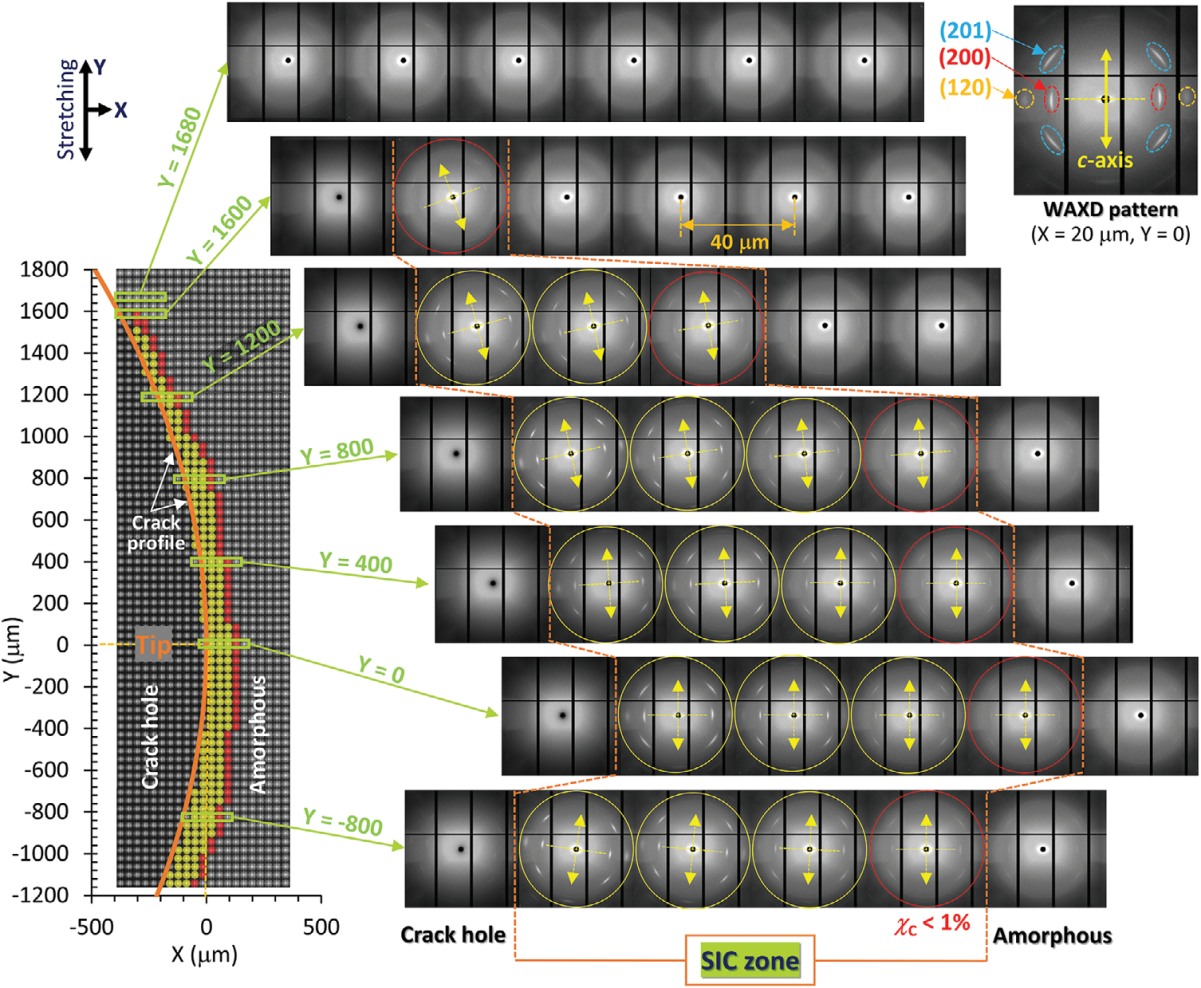
2D mapping of WAXD patterns around the crack tip including peripheral zone. The patterns are mapped on a grid of 21 points on the X‐axis and 75 points on the Y‐axis, each separated by 40 µm. The SIC zone, highlighted in yellow, surrounds the crack‐tip. It is defined by a boundary with <1% crystallinity, marked by red circle patterns. Distinct diffraction spots associate with crystallographic planes, and the orientation of the crystallites (*c*‐axis) are shown by yellow arrow at positions along seven specific lines parallel to the X‐axis. Regions of the crack hole and a totally amorphous state exhibit no crystal diffraction.

The narrow X‐ray beam, with a size of 10 µm  × 8.9 µm, was systematically scanned in the X‐Y plane at intervals of 40 µm around the crack tip which is located at the origin. This approach yielded a map of the 2D WAXD patterns over an area of ≈3000 µm × 800 µm around the crack tip, establishing a definitive basis for characterizing the non‐uniform SIC (**Figure** **2**).

Presence of SIC at each position is uncovered based on the diffractions associated with the crystallographic planes (201), (200), and (120) of NR.^[^ ^9^, ^10^, ^11^, ^12^ ^]^ The crack‐hole area and the totally amorphous regions exhibit no detectable crystal diffraction. The figure picks up the WAXD patterns along specific lines parallel to the X‐axis. At the positions near the crack contour, the crystal diffractions are observed, while the crystal diffraction intensities decay as the positions depart from the crack contour. The SIC region, delineated by yellow patterns, corresponds to the area enclosed between the crack contour and a boundary characterized by a crystallinity of <1%, which is represented by red circle patterns. At the positions in a significantly large distance from the crack contour, they show no crystal diffractions, thereby assigned as the totally amorphous state. The SIC zone is nearly symmetric, and extends ≈160 ± 20 µm along the Y = 0 line, and ≈1600 µm in the Y‐direction away from the crack tip when *ε*_Y,macro_ is 0.62. It is important to recognize that the boundary between SIC and amorphous phases includes inherent ambiguity. This is partly due to the non‐uniform strain distribution near the crack tip, which triggers crystallization and consequently blurs the boundary. In addition, the gradual transition from amorphous to crystalline state—inherent characteristic of SIC in NR—precludes a sudden onset of crystallinity. Instead, crystallinity increases progressively from zero with strain, even during uniform uniaxial stretching.^[^ ^55^, ^56^ ^]^

**Figure 3:**
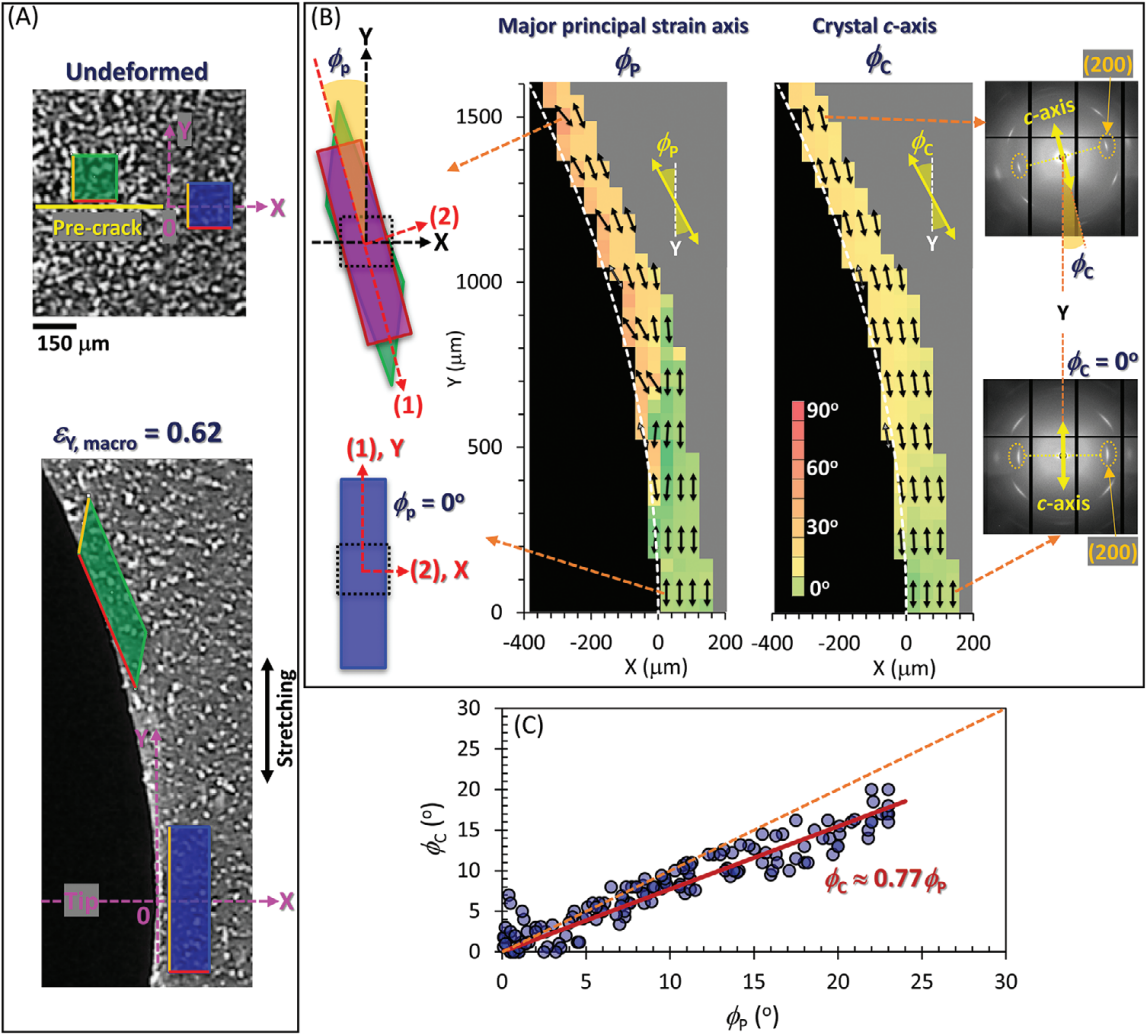
Spatial distributions of local tilting of principal strain axis and crystal *c*‐axis near a crack tip. A) Deformation of a square finite element due to crack‐tip opening at two distinct positions, that is, near and distant from the Y = 0 line. The applied macroscopic tensile true strain is 0.62. A supplementary movie detailing this is available in Supporting Information. Note that the green and blue 150 µm‐squares in the figure and Movie S1 (Supporting Information) serve only for a visual aid to illustrate the local deformations, and not indicative of the resolution of the DIC measurements. B) Spatial distributions of the angles of major principal strain axis (*ϕ*_p_) and crystal *c*‐axis (*ϕ*_c_) relative to the Y‐axis in the vicinity of a crack tip and its peripheral zone. The notations (1) and (2) designate the directions of the major and minor principal strains, respectively, within the local strain tensor. The angle *ϕ*_p_ at each specific position within its spatial distribution is evaluated concurrently at the identical position as the angle *ϕ*_c_ in its respective distribution. To prevent data overlap in the visualization, arrows mark data points spaced at 160 µm intervals, equivalent to every fourth measuring point. C) Plots of *ϕ*_c_ against *ϕ*_p_ for all measured positions. The solid line represents the result by a linear regression with a slope of 0.77, indicating the proportional relationship between *ϕ*_c_ against *ϕ*_p_. The dashed line represents the line with a slope of unity for comparison.

As depicted in Figure 2, both the intensities of the crystal diffractions and the direction of the crystal *c*‐axis exhibit significant variations depending on their positions. These variations in crystal features result from the pronounced non‐uniformity of the strain field, which acts as a trigger for crystallization. The local distortion caused by crack tip opening is complicated and position‐dependent. The non‐uniform strain field is characterized through our micro‐DIC analysis based on the microscope observation of fine speckle patterns. The speckles are ≈20 µm in size, and these patterns were captured at a high resolution, with a pixel size of ≈4 µm on the specimen, as described in Experimental Section. When the crack opens, positions near the Y = 0 line experience stretching along the Y‐axis with slight contraction along the X‐axis, occurring without any body rotation (as indicated by a distortion of blue square in **Figure** **3**A; Movie S1, Supporting Information). In contrast, positions significantly distant from the Y = 0 line (where |Y| >> 0) undergo more intricate displacements including body rotation and shear in the XY plane (as shown by a distortion of green square in Figure 3A; Movie S1, Supporting Information). It should be noted that the green and blue 150 µm‐squares in Figure 3A and Movie S1 (Supporting Information) serve only for a visual aid to illustrate their local deformations, and not indicative of the resolution of the DIC measurements. Crystallization is expected to stem predominantly from the maximum tensile component present in the total displacement associated with the crack tip opening. DIC analysis can evaluate the principal strain tensor at each position, devoid of any diagonal component, enabling discernment of the principal strain axis and its intrinsic strains. As positions deviate from the Y = 0 line, a noticeable tilt in the principal strain axis relative to the Y‐axis becomes apparent (Figure 3B). In contrast, positions

near the Y = 0 line have a principal strain axis parallel to the Y‐axis. Figure 3B highlights a tight correlation between the tilt angles of the principal strain axis (*ϕ*_p_) and the crystal *c*‐axis (*ϕ*_c_) concerning the Y‐axis. This correlation signifies that finite tilting of the crystal *c*‐axis relative to the Y‐axis is a consequence of the tilting of the principal strain axis, which, in turn, reflects finite 2D shear deformation occurring at the corresponding position (detailed in Figure S1C, Supporting Information). A previous study on bulk NR specimens reported a tilt in the crystal *c*‐axis under a uniform deformation composed of uniaxial stretch and subsequent shear.^[^ ^57^ ^]^ Earlier researches on stationary or fatigue cracks in NR^[^ ^12^, ^37^ ^]^ anticipated and confirmed a correlation between *ϕ*_p_ and *ϕ*_c_, albeit achieved with lower resolution using a considerably broader x‐ray beam (≈200 µm square). Our findings demonstrate a pronounced correlation between *ϕ*_p_ and *ϕ*_c_ at a more microscopic level, which is in harmony with these prior findings. The relationship between *ϕ*_p_ and *ϕ*_c_ is quantified in the plots shown in Figure 3C. The data exhibit a linear correlation, represented by a proportional factor of ≈0.77. This indicates that the tilt of the crystal *c*‐axis is somewhat less pronounced than that of the principal strain axis. The complex strain field around crack‐tips is characterized by a combination of uniaxial and substantial shear strains. Consequently, the tilt of the *c*‐axis does not mirror directly the tilt of the principal strain axis. These observations underscore the crucial role of shear deformation in the orientation of SIC microcrystallites.

**Figure 4:**
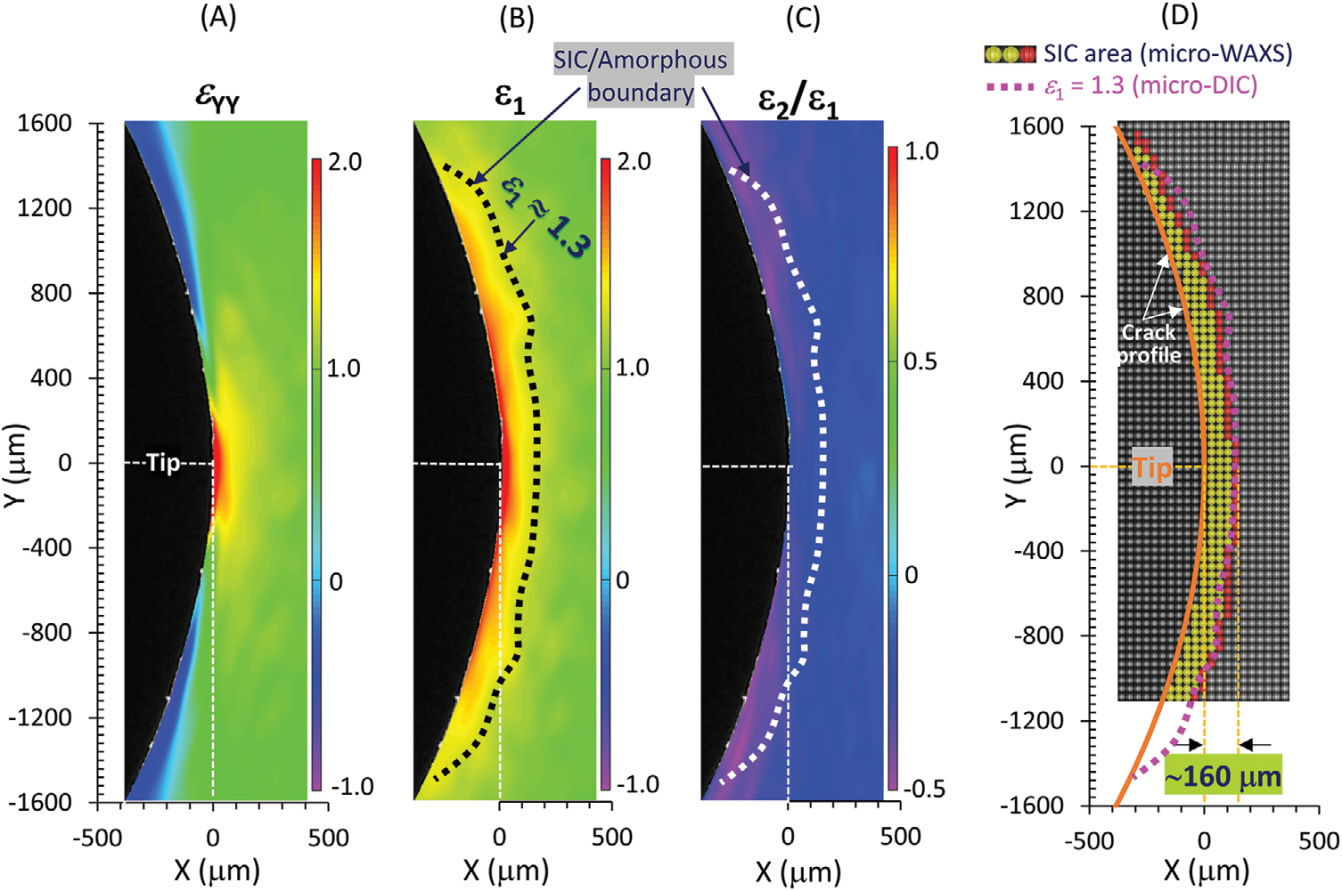
Spatial distribution of local strains around a crack tip. 2D distributions of A) true strain *ε*_yy_ in laboratory coordinate, B) major principal true strain *ε*_1_, and C) strain biaxiality of principal strains *ε*_2_/*ε*_1_ for a pre‐notched NR specimen subjected to an applied macroscopic tensile true strain of 0.62. The dotted line depicts the iso‐*ε*_1_ line of *ε*_1_ = 1.3. D) Comparison between the SIC zone (as shown in Figure 2) identified by micro‐beam WAXD measurements, and the area where *ε*_1_ > 1.3, as determined by micro‐DIC. The iso‐*ε*_1_ line of *ε*_1_ = 1.3 closely parallels the boundary between SIC and amorphous states. The characteristic strain value *ε*_1_ = 1.3 is similar to the SIC onset strains for a corresponding un‐notched NR specimen undergoing planar and uniaxial stretching (Figure S2, Supporting Information).

To correlate the non‐uniform fields of strain and crystallinity, we utilize the major and minor components of the principal strain tensor [*ε*_1_ and *ε*_2_, expressed in true (Hencky) strain, respectively] due to finite *ϕ*_p_, instead of the YY‐ and XX‐components of the strain tensor measured in laboratory coordinates (*ε*_YY_ and *ε*_XX_, respectively). The quantity *ε*_1_ corresponds to the maximum tensile strain involved in the deformation of interest. Near the crack tip, *ε*_1_ exceeds 2 (or nominal strain of 6) due to significantly high strain concentration, even though the applied global true strain (*ε*_Y,macro_) is only 0.62 (or nominal strain of 0.85) (**Figure** **4**B). The major principal strain *ε*_1_ escalates as the position approaches the crack tip, while *ε*_1_ becomes close to the *ε*_Y,macro_ value at the positions in a significantly large distance from the crack tip. Especially in the peripheral regions far from the crack tip—which undergo significant body rotation during crack tip opening (Figure 3A)—a noticeable discrepancy exists between *ε*_1_​ and *ε*_YY_ ​(Figure 4A,B). This considerable difference results from the finite tilting of the principal strain axis (Figure 3B).

It is noteworthy that the iso‐strain line of *ε*_1_ = 1.3 is approximately aligned with the SIC/amorphous boundary as evaluated from the micro‐beam WAXD analysis (Figure 4D). This simplicity is somewhat unexpected but attributed to the limited variation in types of deformation (characterized by the strain biaxiality *ε*_2_/*ε*_1_) within the SIC region. Specifically, the ratio *ε*_2_/*ε*_1_ in the area enclosed by this iso‐*ε*_1_ line varies narrowly, ≈−0.25 ± 0.1, as depicted in Figure 4C. The corresponding deformations are intermediate between planar (*ε*_2_/*ε*_1_ = 0) and uniaxial stretching (*ε*_2_/*ε*_1_  ≈ −0.5). Our WAXS experiments on corresponding unnotched NR specimens reveal that the critical strains for SIC onset under both planar and uniaxial stretching are *ε*_1_ ≈ 1.4 (Figure S2, Supporting Information), closely aligning with the SIC/amorphous boundary condition (*ε*_1_ = 1.3) observed herein. Chen et al. examined the critical strains for SIC onset in unnotched NR sheets under various biaxial stretching conditions.^[^ ^58^ ^]^ They found that the critical major strains for SIC onset, while dependent on the applied biaxial strain ratios, remained almost unchanged between uniaxial and planar stretching. These consistencies suggest that the SIC onset strain condition around crack tips is comparable to those in unnotched specimens under uniform strain, given the relatively narrow range of local deformation types in the SIC area.

**Figure 5:**
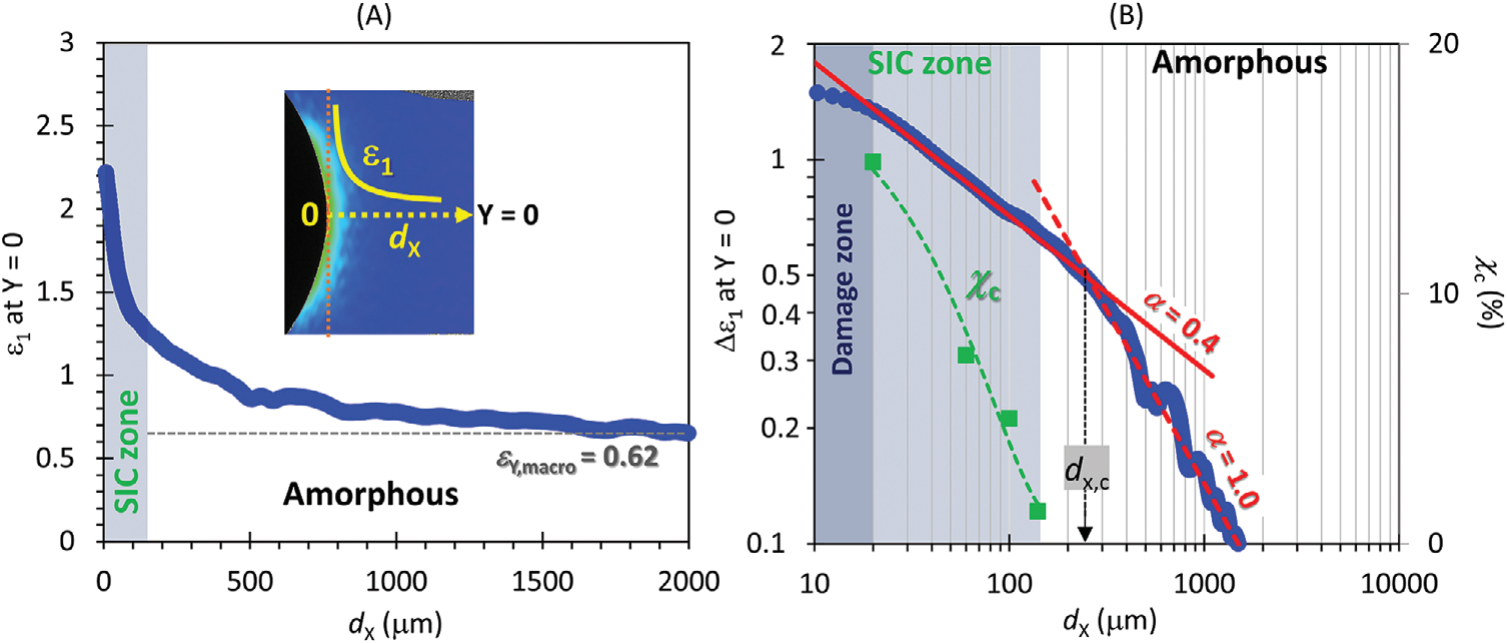
1D distribution of local strain around a crack‐tip along the Y = 0 line. A) Plot of the principal true strain *ε*_1_ against the distance from the crack tip (*d*_x_) for a pre‐notched NR specimen under an applied macroscopic tensile true strain of 0.62, measured along the Y = 0 line. B) Log‐log representation of the true strain increment (Δ*ε*_1_) due to crack tip opening as a function of *d*_x_. Two distinct linear regions, with different slopes, represent the SIC and amorphous zones. The crossover between these zones occurs around *d*_x,c_ = 230 µm. For *d*_x_ < 20 µm, where the dataset deviates from power law behavior, the region corresponds to the damage (or plastic) zone.

Generally, the local vertical strain *ε*_1_ ( = *ε*_YY_) along the Y = 0 line steeply increases as the position approaches the crack tip,^[^ ^59^, ^60^, ^61^, ^62^ ^]^ as observed in this study. This strain singularity behavior is notably influenced by SIC. Strain singularity is assessed through the strain increment resulting from crack tip opening (Δ*ε*_1_), obtained by subtracting the applied global strain from the total strain, Δ*ε*_1_ = *ε*_1_ – *ε*_1,macro_ (**Figure** **5**A). Double logarithmic plots of Δ*ε*_1_ against the distance from the crack tip at Y = 0 (*d*_x_) exhibit an upturn behavior characterized by a power law (Δ*ε*_1_ ∼ *d*_x_^−α^), showing a distinct crossover of *α* at *d*_x,c_  ≈ 230 µm from 0.40 and 1.0. Micro‐beam WAXD analysis (Figure 2) reveals that crystallinity decays as the position departs from the crack tip, with the region at *d*_x_ > 160 µm remaining amorphous. The crossover of *α* reflects the difference in the microstructure of rubber matrix, transitioning from a partially crystallized state with finite crystallinity distribution to a fully amorphous state. Notably, the position *d*_x,c_ aligns closely with the SIC/amorphous boundary (*d*_x_ ≈ 160 µm) as determined by micro‐beam WAXD analysis. A noticeable downward deviation from the power law behavior has been observed at positions extremely close to the crack tip (*d*_x_ < 20 µm). This region is likely attributed to the influence within the damage or plastic zone,^[^ ^33^, ^34^, ^62^ ^]^ where the rupture of polymer networks^[^ ^34^, ^63^ ^]^ and the formation of nano‐cavitation^[^ ^50^, ^64^ ^]^ predominates. The size of the observed damage zone is comparable to those reported in the studies using small angle X‐ray scattering.^[^ ^34^, ^50^ ^]^

**Figure 6:**
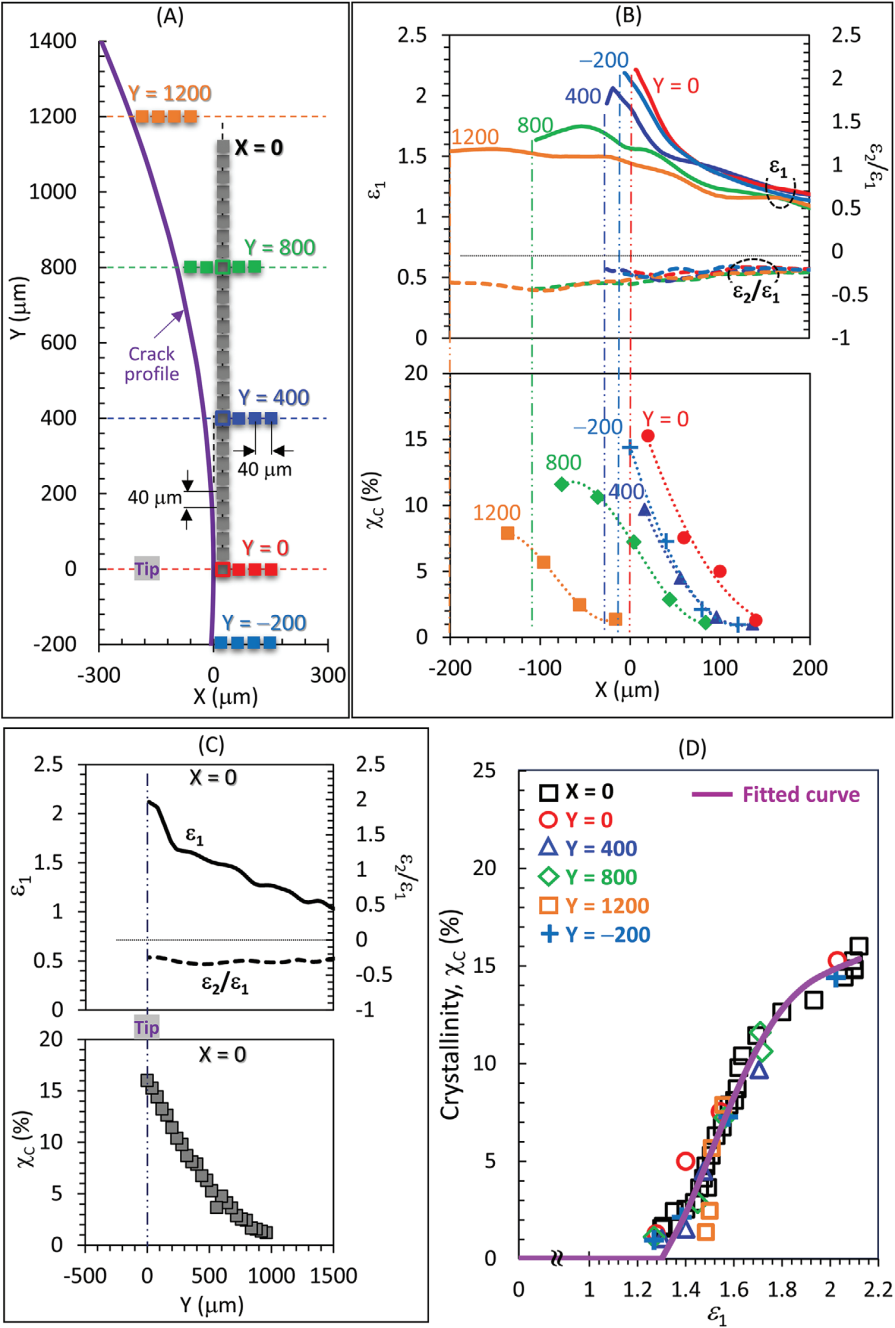
Correlations between local strains and local crystallinity in the SIC zone. Correlations between major principal true strain (*ε*_1_), strain biaxiality (*ε*_2_/*ε*_1_), and local crystallinity (*χ_c_*) at various positions along A) specific six lines: B) the Y = 0, 400, 800, 1200, and −200 µm lines, and C) the X = 0 line. D) Plots of *χ*_c_ against *ε*_1_ for the data in (B) and (C). The various data at different positions fall on a singular fitted curve, depicted by the violet line. The fitted curve is given by a quartic function, *χ*_c_ (X,Y) = ∑ *a_k_* [*ε*_1_ −*ε*_1_*]*^k^* for *ε*_1_≥*ε*_1_* with *k* = 1, 2, 3,4, and *χ*_c_ (X,Y) = 0 for *ε*_1_<*ε*_1_*. The SIC onset strain *ε*_1_* is set at 1.3, and the coefficients from the fitting are *a*_1_ = 17.85, *a*_2_ = 70.02, *a*_3_ = −148.55, and *a*_4_ = 78.62.

The distribution of degree of crystallinity (*χ*_c_) near the crack tip has been assessed by analyzing 2D WAXD patterns at various positions. The method for evaluating *χ*_c_ is detailed in Figure S3A (Supporting Information). We investigated the correlations of *χ*_c_ with *ε*_1_ and *ε*_2_/*ε*_1_ at different positions on six specific lines (**Figure** **6**A–C). It was observed again that the strain biaxiallity (*ε*_2_/*ε*_1_) within the SIC zone is approximately constant (≈−0.25), irrespective of positions on all lines. Ahead of the crack tip (X > 0) and on the vertical line from the crack tip (X = 0), *ε*_1_ increases steeply as either X or Y approaches zero. Behind the crack tip (X < 0), in the range 0 < X < −40 µm, the *ε*_1_ values at each line either decrease or exhibit a quasi‐plateau when departing away from the crack tip. For X < −40 µm, *ε*_1_ at each line shows a mild dependence on X showing a broad maximum peak. Generally, *χ*_c_ increases as the position approaches the crack contour.

Notably, the *χ*_c_ data for various positions in Figure 6A predominantly align on a single line (depicted by a violet line) when plotted against *ε*_1_ at the corresponding positions (Figure 6D). This outcome indicates that the non‐uniform distribution of *χ*_c_ around the crack tip can be accurately approximated using a function solely dependent on *ε*_1_, irrespective of *ε*_2_. The violet line represents a fitted quartic function of *ε*_1_ (X,Y) for *χ*_c_ at the respective position:

(1) $$\begin{array}{c} \chi_{c}\left(X,Y\right)={\sum a_{k}\left[\varepsilon_{1}\left(X,Y\right)-\varepsilon_{1}*\right]}^{k}\;\;with\;\;k=1,\;2,\;3,\;4\;\;\;\\ when\;\varepsilon_{1}>\varepsilon_{1}*;\chi_{c}\left(X,Y\right)=\;\;0\;\;at\;\varepsilon_{1}<\varepsilon_{1}* \end{array}$$

Where *ε*_1_* is the *ε*_1_ value at the SIC/amorphous boundary and set at 1.3 here, and the fitted *a_k_* values are indicated in the caption of Figure 6D. This characteristic simplicity of *χ*_c_, based solely on *ε*_1_, is consistent with the approximation of the SIC/amorphous boundary by the iso‐*ε*_1_ line (Figure 4D). Importantly, no master function for *χ*_c_ analogous to Equation (1) can be obtained using *ε*_YY_ measured in the laboratory coordinate, as demonstrated in Figure S4 (Supporting Information). This result confirms that *χ*_c_ is governed by the maximum tensile local strain derived in the principal axes coordinates, in other words, the local strain almost along the local crystal *c*‐axis. Equation (1) serves as a phenomenological model to represent the experimentally obtained master relationship between *χ*_c_ and *ε*_1_. Theoretical advancement to elucidate the underlying physics is needed. Currently, there is no established physical model that quantitatively describe the relationship between *χ*_c_ and strain in SIC systems.

**Figure 7:**
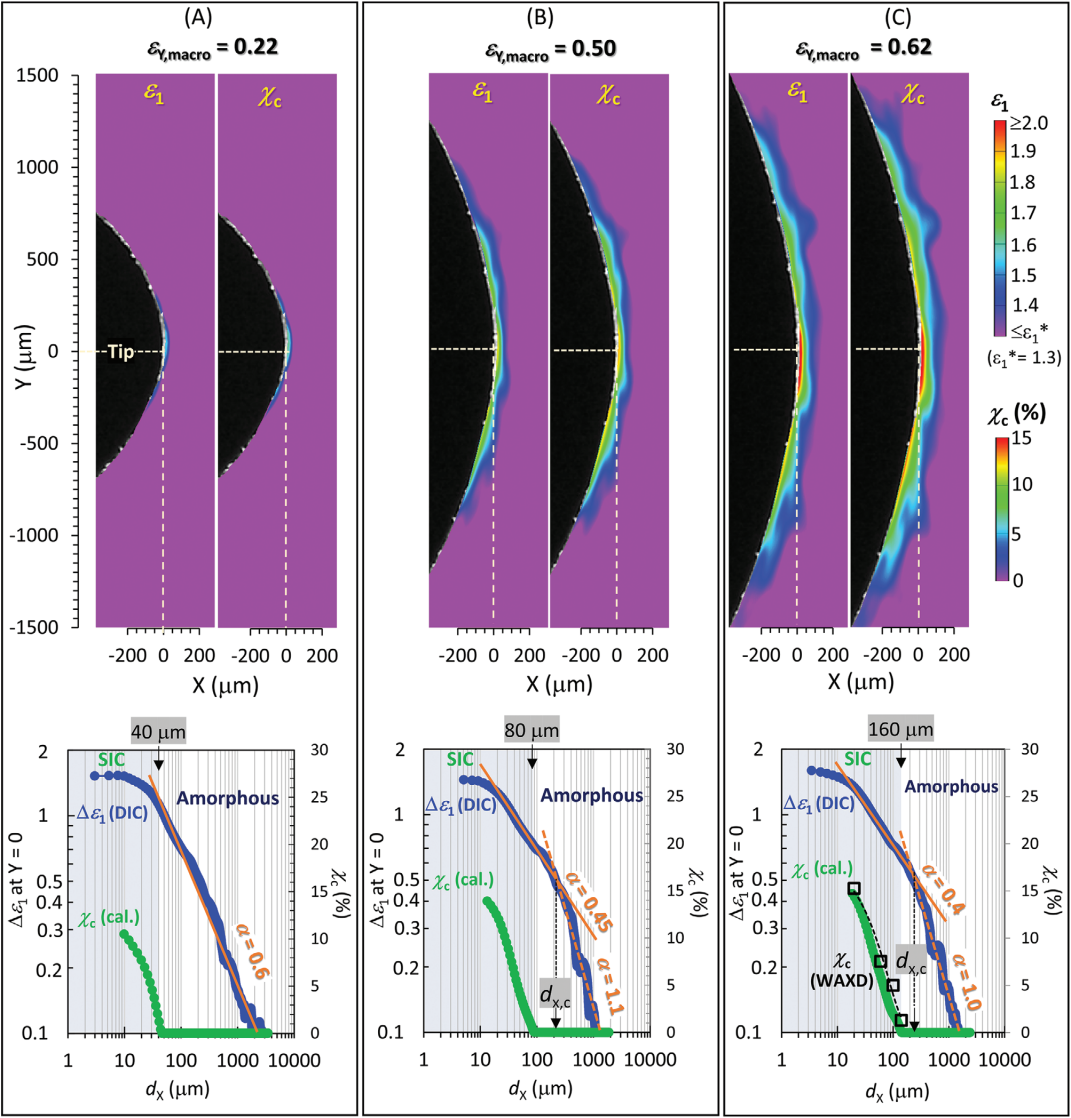
Computed 2D distributions of *χ*_c_ at various degrees of crack tip opening. The applied macroscopic tensile true strain (*ε*_Y,macro_) is A) 0.22, B) 0.50, and C) 0.62. In each panel, the upper right figure showcases the distribution of *χ*_c_ computed using Equation (1) with the *ε*_1_ field data (displayed in the upper left figure) obtained via DIC. The bottom figure presents log‐log plots of the data of Δ*ε*_1_ ( = *ε*_1_ − *ε*_Y,macro_) and the computed *χ*_c_ as functions of *d*_x_ along the Y = 0 line for each *ε*_Y,macro_. As *ε*_Y,macro_ increases, a distinct SIC region (*χ*_c_ > 0) emerges at *ε*_1_ > *ε*_1_* = 1.3, and the crossover point for the *d*_x_ dependence of Δ*ε*_1_ around the boundary between SIC and amorphous regions shifts to larger *d*_x_.

Significantly, Equation (1) facilitates the conversion of the *ε*_1_ field data into a 2D distribution of *χ*_c_, despite its phenomenological nature. This equation allows the computation of both the magnitude of *χ*_c_ and the 2D distribution of *χ*_c_ around the crack tip at any degree of crack tip opening, solely utilizing the corresponding *ε*_1_ field data. **Figure** **7**A–C showcases the evolution of SIC during the crack tip opening at three different applied tensile strain magnitudes (*ε*_Y,macro_), as calculated with the respective *ε*_1_ field data obtained through micro‐DIC. The maximum *ε*_Y,macro_ value in the figure aligns with the value used in the experiment which is close to but below the critical value for initiating crack growth. The computed *χ*_c_ field at each *ε*_Y,macro_ vividly illustrates SIC characteristics around the crack tip, including a non‐uniform *χ*_c_ distribution and the SIC/amorphous boundary. The *χ*_c_ values increase as either X or Y approaches zero, exhibiting a symmetrical distribution with the highest crystallinity found near the crack tip (X = Y = 0). In the figures, calculated *χ*_c_ values in the damage zone at X < 20 µm are omitted, as the experimental data did not elucidate the SIC behavior in this zone.

Equation (1) gives insights into SIC evolution driven by crack tip opening, which is characterized by an increasing *χ*_c_ and a broadening distribution of *χ*_c_. Figure 7A–C also presents the 1D distributions of Δ*ε*_1_ and *χ*_c_ along the Y = 0 line at each *ε*_Y,macro_ value. With increasing *ε*_Y,macro_ (i.e., as the crack opens), a pronounced SIC area emerges. This is obvious by the shifting of the SIC/amorphous boundary (given by the iso‐*ε*_1_ line of *ε*_1_ = 1.3) toward larger *d*_x_ values. At smaller crack tip opening, there is no observable crossover in the *d*_x_ dependence of Δ*ε*_1_ for the micro‐DIC data, with the calculated SIC zone confined at *d*_x_ < 40 µm (Figure 7A). For larger openings (Figure 7B,C), the emergence of a distinct SIC zone results in observable crossovers in strain singularity behavior. The crossover position (*d*_x,c_) for *α* moves away from the crack tip as the opening enlarges. This movement correlates with the expansion of the computed SIC zone and a corresponding shift in the SIC/amorphous boundary. While the mechanical behavior of semi‐crystalline polymers—composed of crystalline and amorphous phases—is complex due to their hierarchical structures, degree of crystallinity critically influences their mechanical traits.^[^ ^65^ ^]^ Our finding is crucial for the development of predictive models that can simulate SIC behavior near crack tips in soft materials—a key determinant of mechanical reinforcement.

In the context of engineering application, understanding the influence of filler introduction on SIC is vital, given that filler‐reinforced NR is commonly used for rubber products. Previous studies^[^ ^53^, ^66^, ^67^, ^68^ ^]^ have indicated that the interaction of rubber with fillers reduced the critical strain for SIC onset in comparison to unfilled NR. It is anticipated that the presence of fillers will also notably alter the effects of strain biaxiality on SIC, diverging from the behavior seen in unfilled NR. Future research will uncover the essential role of filler‐rubber interaction in SIC around crack‐tips.

## 3 Conclusion

The present study elucidates the complex relationship between the non‐uniform strain field and SIC in the vicinity of a crack tip and its peripheral zone in notched NR under constant imposed stretch. In peripheral zone apart from the crack tip, the local principal strain axis undergoes tilting relative to the direction of imposed stretch due to the significant influence of crack tip opening. Correspondingly, the *c*‐axis of the resultant SIC crystal also exhibits tilting. The tilt angle of the *c*‐axis is somewhat less pronounced than that of the principal strain axis, while they exhibit a linear correlation. Local crystallinity (*χ*_c_) and the initiation of SIC near the crack tip and its peripheral zones are predominantly influenced by the maximum tensile true strain (*ε*_1_) within the tensor of local principal strains, devoid of diagonal components. The boundary separating SIC and amorphous regions is approximated by the iso‐*ε*_1_ line of *ε*_1_ = 1.3, and the spatial distributions of *χ*_c_ can be described using a function dependent solely on *ε*_1_. This simplicity arises due to the limited variation in types of deformation (characterized by a narrow *ε*_2_/*ε*_1_ range of ≈ −0.25 ± 0.1) within the SIC zone, with corresponding deformations falling between planar and uniaxial stretching. This study paves the way for predicting the heterogeneous spatial distribution of *χ*_c_ during crack tip opening using only the respective strain‐field data.

## 4 Experimental Section

### Materials

The NR specimens were prepared using NR gum (Ribbed Smoked Sheets RSS#3). Vulcanization was conducted by using sulfur (1.4 g per 100 g of rubber) as a cross‐linking agent. The information of the materials, additives, compositions, and preparation procedures is given in Table S1 (Supporting Information).

### Characterization of Strain Field near Crack tip

For the characterization of local crack tip strain field, a micro‐DIC technique (Figure 1B) was employed that quantified the 2D displacement of the micro‐speckle patterns on the specimen surface caused by crack tip opening. A sheet specimen was used with a dimension of 40 mm × 10 mm × 0.25 mm with a 10 mm pre‐crack (notch) along the X‐axis, positioned centrally on one side of the edge (Figure 1A). A random speckle pattern with a typical speckle size of 20 µm was applied on the specimen surface using a high‐pressure spray gun and a water‐based acrylic white ink (GSI Creos H11), as shown in Figure 1B. This labeling process does not alter the mechanical properties of the specimens, as NR is not water‐absorbent.

The specimen was stretched at 25 °C in the Y‐axis with a true strain of 0.62 (nominal strain of 0.85) that was below the critical strain for crack growth initiation (≈0.79 and 1.2 in true and nominal strain, respectively), using a custom‐built manually controllable stretching device. Prior to the measurement, the sample underwent several preliminary loading‐unloading cycles, with the maximum strain matching that used in the DIC/WAXD tests. This preconditioning was designed to remove strain‐hysteresis effect on stress. Microscopy observation verified the consistency of the crack‐tip profile under identical macroscopic tensile strain condition, for the repetition of loading‐unloading cycles.

A set of micro‐speckle images with the resolution of 1200 pixels × 1600 pixels (pixel size of ≈4 µm) in the stretching process was captured by a digital microscope (Hirox KH‐8700), aided by a high‐power LED light source (UFLS‐75, U‐Technology). The image at each applied strain was captured after the stress reached the quasi‐equilibrium state, that is, 10 min after the destination strain was attained (Figure S5, Supporting Information).

The distribution of 2D displacement gradient tensor that characterizes the non‐uniform strain field was evaluated by analyzing the captured speckle images using the VIC‐2D software (Correlated Solutions).^[^ ^69^ ^]^ The 2D local strain tensor at each position measured in laboratory (X, Y) coordinates was calculated from the corresponding displacement gradient tensor.^[^ ^54^ ^]^ The DIC analysis also identifies the major and minor principal strains (*ε*_1_ and *ε*_2_, respectively, in true strain) both in magnitudes and directions.^[^ ^54^ ^]^ The DIC measurement is detailed in Section S1 (Supporting Information).

### Characterization of SIC near Crack‐tip

For the characterization of SIC near the crack‐tip, a micro‐beam WAXD experiment (Figure 1A) was carried out at the BL‐15A2 beamline of the Photon Factory at the High Energy Accelerator Research Organization (Tsukuba, Ibaraki, Japan). The wavelength and size of the x‐ray beam are 0.118 nm and x × y = 10 × 8.9 µm^2^. PILATUS 2 M (DECTRIS Ltd., Baden, Switzerland) was used as a 2D detector. The sample‐detector distance was 200 mm.

The exact same pre‐cracked sample and same custom‐made tensile instrument used in the micro‐DIC experiment were used in the micro‐beam WAXD measurement, thereby enabling the precise correlation between the results of the two independent experiments. The speckle patterns on the specimen surface for micro‐DIC were wiped out before the WAXD measurements. As in the case of the micro‐DIC measurements, the pre‐cracked sample was exposed to a tensile true strain of 0.62 using the same stretching device at 25 °C. The crack contour of the stretched sample was identical with that in the micro‐DIC measurement, confirming the same strain field in the two independent tests. After the stress reached the quasi‐equilibrium after 10 min, the narrow x‐ray beam was irradiated for 0.2 s at each point and scanned over a region around the crack tip, covering a grid of 20 points along the x‐axis and 72 points on the y‐axis, with each point separated by a distance of 40 µm (Figure S6, Supporting Information). The exposure time was set to 0.2 s per point based on preliminary tests that showed this duration minimizes radiation damage while maintaining crystal diffraction patterns during continuous exposure.

1D WAXD profiles were obtained by averaging sectors of the 2D WAXD pattern over an azimuthal angle range of 180^o^ ± 10^o^ where the azimuthal angle was defined in relation to the direction orthogonal to the local extension direction (Figure S3B, Supporting Information). These 1D WAXD profiles were corrected for air scattering contributions prior to calculating crystallinity. Given the specimen thickness of 0.25 mm in its undeformed state, any decrease in scattering intensity due to X‐ray beam absorption was not considered. Computational peak decomposition for each 1D WAXD profile was conducted within the 4–23 nm^−1^ range, employing Lorentzian peak assumptions for the crystalline reflection peaks observed at *q* = 10.0, 12.7, 14.4, and 18.4 nm^−1^. These are assigned to the (200), (201), (120, 002), and (202) reflections, respectively. The crystallinity (*χ*_c_) was determined based on the relation *χ*_c_ = *I*_c_ / (*I*_c_+ *I*_m_), where *I*_c_ represents the total areas of all crystalline peaks and *I*_m_ refers to the amorphous peak (Figure S3A in Supporting Information). This evaluation method for *χ*_c_ inherently compensates for thickness variation although finite variations in sample thickness are expected near the edge of the crack, because both *I*_c_ and *I*_m_ are proportionally influenced by changes in thickness. Consequently, this proportional change ensures that the resultant *χ*_c_ value remains unaffected by thickness.

## Conflict of Interest

The authors declare no conflict of interest.

## Supporting information

Supporting Information

Supplemental Movie 1

## Data Availability

The data that support the findings of this study are available from the corresponding author upon reasonable request.
