# Supplementary material for: Unraveling Non‐Uniform Strain‐Induced Crystallization Near a Crack Tip in Natural Rubber
Source: Adv Sci (Weinh). 2024 Jan 16;11(12):2307741. doi: 10.1002/advs.202307741 (PMC10966571; doi:10.1002/advs.202307741)
Supplement: Supplementary file 1 — Supporting Information [file ADVS-11-2307741-s001.pdf]

ADVANCED SCIENCE
Open Access

Supporting Information

for *Adv. Sci.*, DOI 10.1002/advs.202307741

Unraveling Non-Uniform Strain-Induced Crystallization Near a Crack Tip in Natural Rubber

Thanh-Tam Mai, Tomohiro Yasui, Ruito Tanaka, Hiroyasu Masunaga, Taizo Kabe, Katsuhiko Tsunoda, Shinichi Sakurai and Kenji Urayama**

Supporting Information

Unraveling Non-uniform Strain-induced Crystallization Near a Crack Tip in Natural Rubber

Thanh-Tam Mai†1, Tomohiro Yasui†2, Ruito Tanaka[2], Hiroyasu Masunaga[3], Taizo Kabe[3], Katsuhiko Tsunoda[4], Shinichi Sakurai*2 and Kenji Urayama*1

[1]*Graduate School of Engineering, Department of Material Chemistry, Kyoto University, Nishikyo-ku, Kyoto 615-8510, Japan*

[2] *Department of Biobased Materials Science, Kyoto Institute of Technology, Kyoto 606-8585, Japan*

[3]*SPring-8, Hyogo, Japan*

[4] *Sustainable and Advanced Materials Division, Bridgestone Corporation, Tokyo 187-8531, Japan*

†Two authors contributed equally as the first authors.

*Corresponding authors: Email: urayama.kenji.2s@kyoto-u.ac.jp (K.U.); shin@kit.ac.jp (S.S.)

1. Micro-DIC measurements

In DIC measurement, an undeformed micro-speckle image served as the reference image with a global orthogonal coordinate system of X and Y (**Figure 1B**). The 2D displacement gradient tensor, denoted as f_{ij} (i,j = X,Y), was calculated using the VIC-2D® system (Correlated Solutions) − a comprehensive solution for DIC measurements on the specimens.

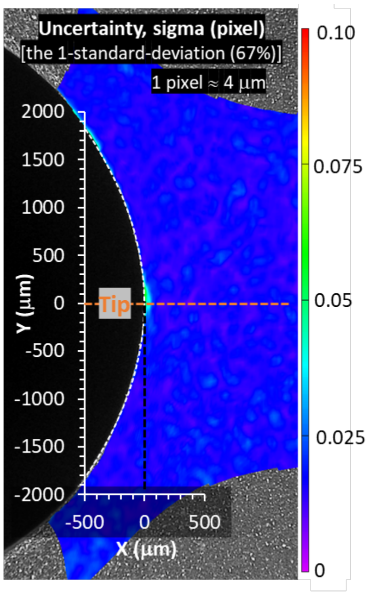

Figure S1A. The 1-standard deviation confidence (Sigma) in the match around the crack-tip of the DIC measurement.

Figure S1A illustrates displacement accuracy (Sigma [pixels]), representing the 1-standard deviation confidence in the match around the crack-tip in the DIC measurement. A Sigma value of zero indicates a perfect match, while higher Sigma values suggest noise, excessive gradients, or a potential match failure. In this context, the Sigma values are less than 0.05 pixel or 0.2 pixel (with a pixel size of about 4 micrometers). This implies that the matching error in the displacement measurements is sufficiently small for the data used in the discussion, ensuring the reliability of the results. The values of f_{ij}, encompassing f_{XX}, f_{YY}, and f_{XY} (or f_{YX}), represent the X-axis, Y-axis, and shear components respectively, and are provided in **Figure S1B**. The 2D local strain tensor was empirically derived from the displacement gradient tensor. The true (Hencky) strain tensor (ε_{ij}, where i,j = X,Y) and the corresponding principal strains (ε_1 and ε_2) were computed. ε_{XX} and ε_{YY} signify true strain along the X and Y axes, respectively, measured in laboratory coordinate, while $\varepsilon_{XY} = \varepsilon_{YX}$ denote tensorial shear strains (**Figures 4** and **S1C**). The principal true strains are the maximum (ε_1, major strain) and minimum (ε_2, minor strain) normal strain values determined according to the local principal coordinate system (1 and 2) wherein the shear strain value is zero (**Figures 4** and **S1C**). This local principal coordinate system in the deformed state is generated by a rotation from the global coordinates in the reference (undeformed) state, defined by a rotation angle ϕ_p. In this study, ϕ_p, in the vicinity of the crack-tip, includes the body rotation resulting from crack-tip openings (**Movie S1** in SI).

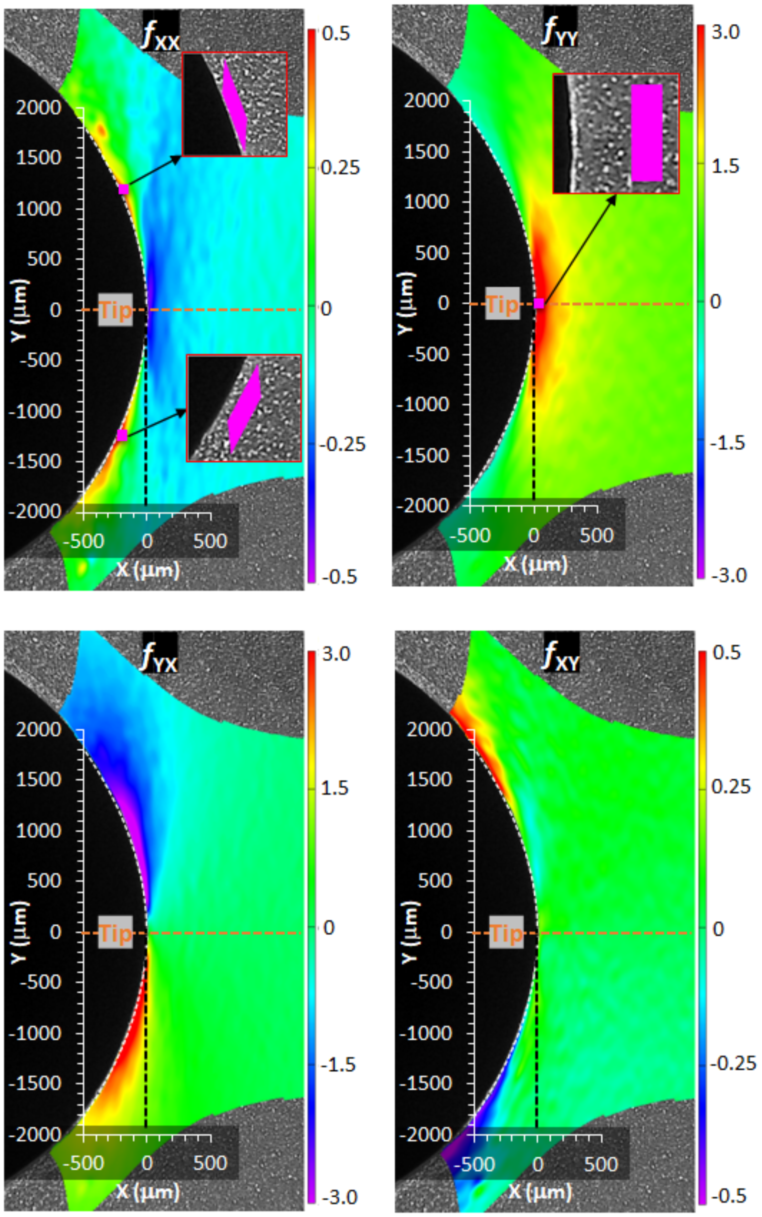

Figure S1B. 2D displacement gradient tensor. The computed components of the 2D displacement gradient tensor f_{ij} (where i,j = X,Y), which represent the X-axis (f_{XX}), y-axis (f_{YY}), and shear (f_{XY} and f_{YX}) components respectively, obtained from micro-DIC measurements for a notched NR specimen under constant imposed stretch.

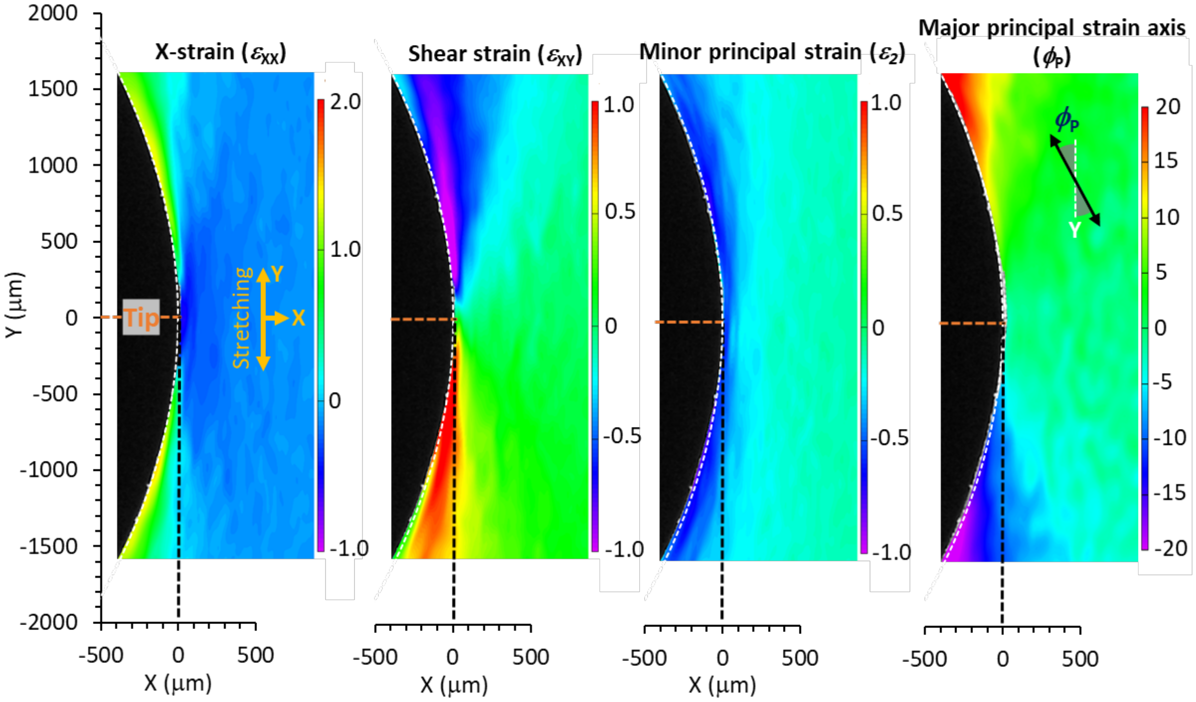

Figure S1C. Strain fields in the X-axis (ε_{XX}), shear strain (ε_{XY}), the minor principal strain (ε_2), and major principal axis.

2. WAXD measurement for unnotched NR specimen

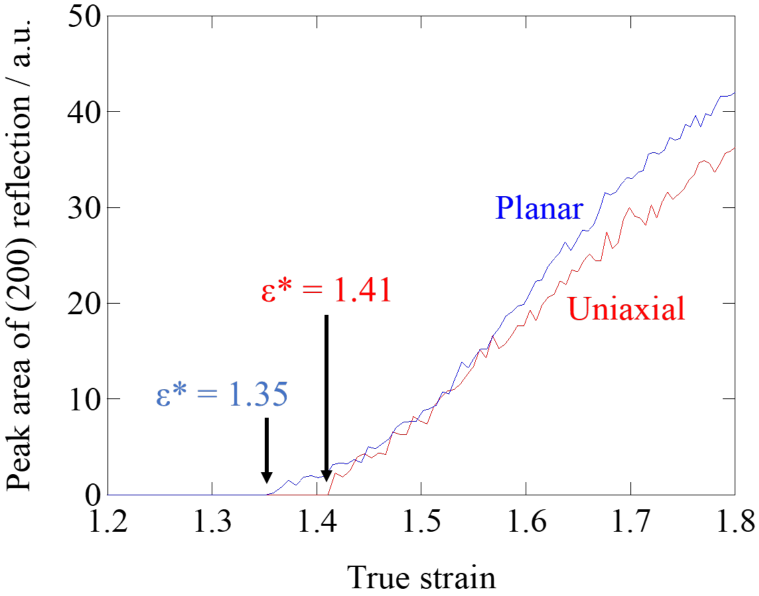

Figure S2. Peak area of (200) reflection as a function of imposed true strain in planar and uniaxial stretching for unnotched NR specimens at 25 °C. The true strain for the onset of SIC in each stretching type is denoted by ε^*. Planar stretching was achieved by stretching in the Y-direction using a wide sheet specimen with a dimension of 90(X) mm × 10(Y) mm × 0.25(Z) mm. Uniaxial stretching was conducted by stretching in y-direction using a strip specimen with a dimension of 5(X) mm × 30(Y) mm × 0.25(Z) mm.

3. Evaluation of crystallinity, χ_c

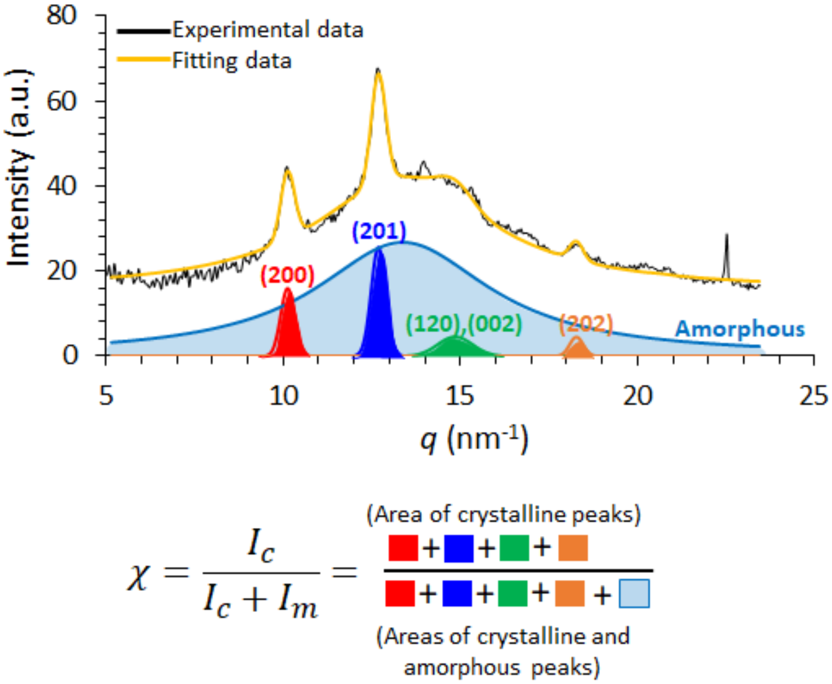

$$\chi = \frac{I_c}{I_c + I_m} =$$

Figure S3A. Determination of crystallinity from 1D WAXD profiles. Demonstrates the computational peak decomposition process used to derive crystallinity (χ_c). The figure highlights the defined Lorentzian peaks corresponding to various crystalline reflections, as well as the amorphous peak. χ_c is computed from the total areas of all crystalline peaks (I_c) and the area under the amorphous peak (I_m) using the relation $\chi = I_c / (I_c + I_m)$.

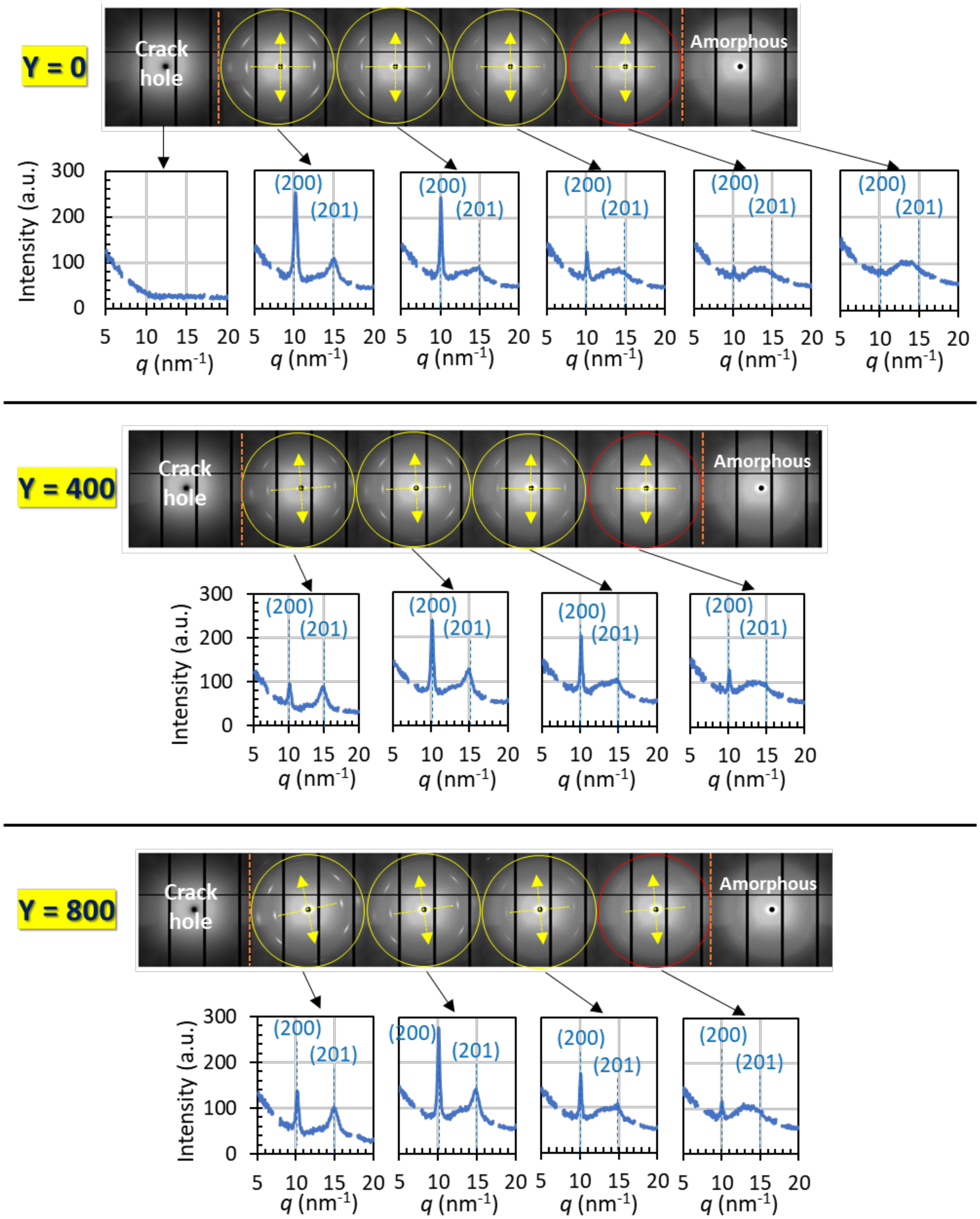

Figure S3B. The 1D WAXD profiles obtained from the corresponding 2D WAXD patterns at several positions along the lines of Y = 0, 400, and 800. Each 1D WAXD profile was obtained by averaging the sectors of the corresponding 2D WAXD pattern over an azimuthal angle range of 180° ± 10°. The azimuthal angle was defined in relation to the direction orthogonal to the local extension direction. These 1D WAXD profiles were corrected for air scattering contributions prior to calculating crystallinity.

4. Plots of χ_c versus ε_{YY}

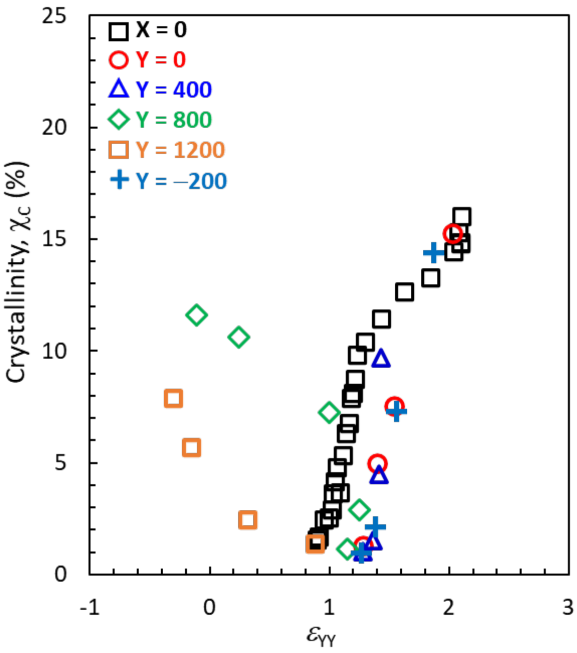

Figure S4. Plots of local crystallinity (χ_c) against true strain ε_{YY} measured in laboratory coordinate at respective positions. No master function for χ_c is obtained, in contrast to the corresponding plots against principal true strain ε_1 (Figure 6D).

5. Stress-relaxation behavior of NR sample

Figure S5 illustrates the nominal stress (σ) over time for a NR specimen, following the application of a constant tensile true strain of 1.94. A wide NR sheet specimen (40 mm × 10 mm × 0.25 mm) was uniaxially stretched to a true strain of 1.94 (equivalent to a nominal strain of 6.0), using a constant crosshead speed of 15 mm s^{-1}, which corresponds to an initial strain rate of 1.5 s^{-1}. Subsequently, the decay of the tensile force was monitored over time. The data indicate a finite stress relaxation (approximately 30%), reaching a quasi-equilibrium state within 10 min. Measurements of the μ-DIC and the μ-beam scanning WAXD on the notched NR specimen were conducted 10 min after the application of tensile strain, ensuring that the effects of stress relaxation effect were largely mitigated in these measurements.

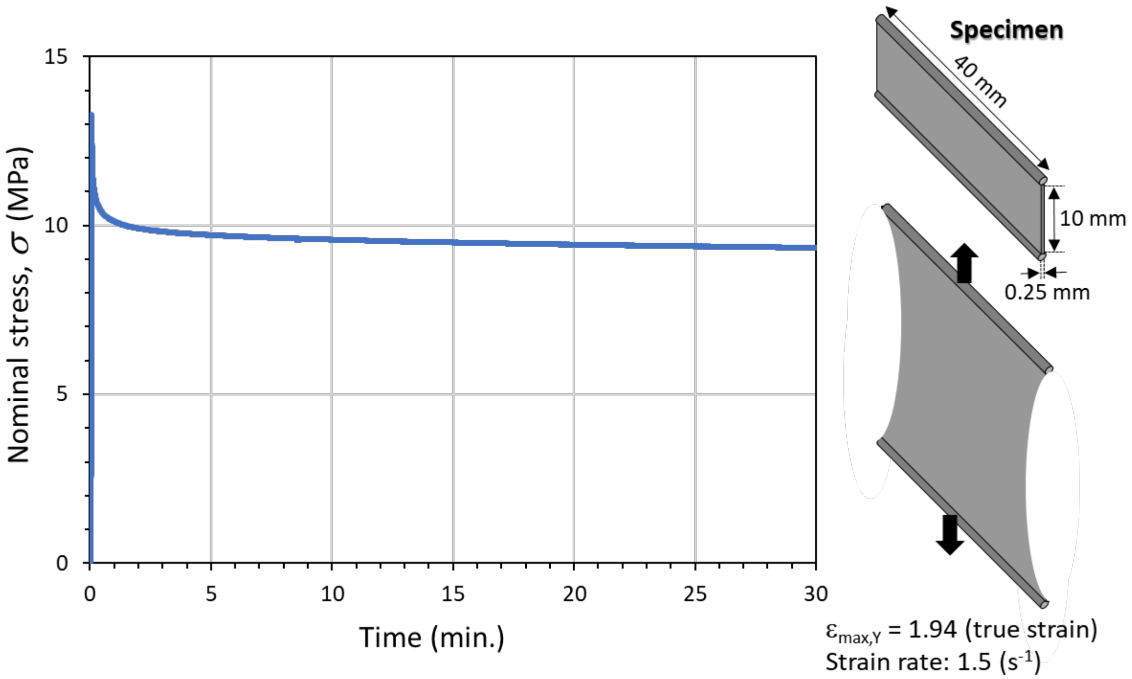

Figure S5. The normalized stress over time for a NR specimen, following the application of a constant true strain of 1.94. The stress reaches a quasi-equilibrium state within 10 min.

6. Sample preparation

Table S1. Composition of rubber specimens

	**NR**
Natural rubber gum [a)]	100 g
Sulfur [b)]	1.5 g
Stearic acid	2 g
Zinc oxide	5 g
TMDQ [c)]	0.3 g
6PPD [d)]	1 g
CBS [e)]	1.5 g

[a)] Ribbed Smoked Sheets RSS#3
[b)] Sanfel-EX Sanshin Chemical Co.
[c)] Polymerized 2,2,4-trimethyl-1,2-dihydro-quinoline
[d)] N-1,3-dimethylbutyl-N'-phenyl-*p*-phenylenediamine
[e)] N-cyclohexyl-2-benzothiazole sulfenamide

Steraric acid, zinc oxide (ZnO) and CBS were employed as vulcanization accelerator, and TMQD and 6PPD were used as anti-aging agent. Surfur was used as cross-linker. Firstly, the mixture of natural rubber gum, stearic acid, ZnO, TMQD, and 6PPD was subjected to shear for 2 min in a chamber at 80 ^oC. Subsequently, CBS and sulfur were added to the mixture for vulcanization, and the mixture was sheared for 1.5 min at 80 ^oC. The mixture underwent further shear using an open-roll mill for 5 min at 60 ^oC. Specimen sheets were made by hot press technique at 160 ^oC for 8 min.

7. Scanning micro-beam WAXD measurement

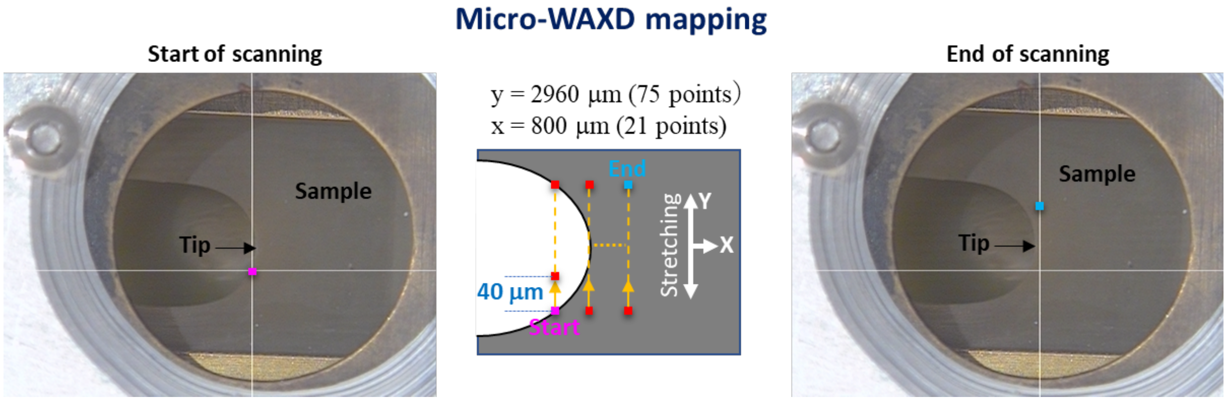

Figure S6. The real-time micro-beam WAXD scanning at applied true strain $\varepsilon_{y,macro}$ = 0.62 (or nominal strain of 0.85) in the vicinity of the crack-tip. The grid, used for the mapping of strain-induced crystallization, consists of 21 points along the x-axis and 75 points on the y-axis, with each point separated by a distance of 40 µm. A narrow x-ray beam with a wavelength of 0.118 nm and a dimension of x × y = 10 × 8.9 μm^2 was irradicated for 0.2 seconds at each point.
